# E-Selectin/AAV2/2 Gene Therapy Alters Angiogenesis and Inflammatory Gene Profiles in Mouse Gangrene Model

**DOI:** 10.3389/fcvm.2022.929466

**Published:** 2022-06-16

**Authors:** Antoine J. Ribieras, Yulexi Y. Ortiz, Yan Li, Carlos T. Huerta, Nga Le, Hongwei Shao, Roberto I. Vazquez-Padron, Zhao-Jun Liu, Omaida C. Velazquez

**Affiliations:** ^1^Division of Vascular Surgery, DeWitt Daughtry Family Department of Surgery, University of Miami Miller School of Medicine, Miami, FL, United States; ^2^Vascular Biology Institute, University of Miami Miller School of Medicine, Miami, FL, United States

**Keywords:** E-selectin (CD62E), gene therapy, angiogenesis, chronic limb-threatening ischemia, peripheral artery disease, gangrene

## Abstract

For patients with chronic limb-threatening ischemia and limited revascularization options, alternate means for therapeutic angiogenesis and limb salvage are needed. E-selectin is a cell adhesion molecule that is critical for inflammation and neovascularization in areas of wound healing and ischemia. Here, we tested the efficacy of modifying ischemic limb tissue by intramuscular administration of E-selectin/AAV2/2 (adeno-associated virus serotype 2/2) to modulate angiogenic and inflammatory responses in a murine hindlimb gangrene model. Limb appearance, reperfusion, and functional recovery were assessed for 3 weeks after induction of ischemia. Mice receiving E-selectin/AAV2/2 gene therapy had reduced gangrene severity, increased limb and footpad perfusion, enhanced recruitment of endothelial progenitor cells, and improved performance on treadmill testing compared to control group. Histologically, E-selectin/AAV2/2 gene therapy was associated with increased vascularity and preserved myofiber integrity. E-selectin/AAV2/2 gene therapy also upregulated a panel of pro-angiogenic genes yet downregulated another group of genes associated with the inflammatory response. This novel gene therapy did not induce adverse effects on coagulability, or hematologic, hepatic, and renal function. Our findings highlight the potential of E-selectin/AAV2/2 gene therapy for improving limb perfusion and function in patients with chronic limb-threatening ischemia.

## Introduction

Peripheral artery disease (PAD) affects 200 million people globally, up to 10% of whom suffer from its most severe form, chronic limb-threatening ischemia (CLTI) (1). CLTI is characterized by hemodynamic compromise to the lower extremities leading to ischemic rest pain, gangrene, or non-healing wounds (2). It is a condition associated with decreased quality of life, increased risk for amputation, and increased 5-year mortality (2). Beyond risk factor optimization (i.e., smoking cessation, lipid-lowering therapy, glycemic control), surgical revascularization with open bypass or endovascular intervention remains the cornerstone of therapy (3). However, these therapies often require repeated procedures, and many patients still suffer limb loss to amputation. For the 30% of CLTI patients who are not candidates for or have failed prior revascularization attempts, alternate methods are needed to improve perfusion to the extremities (1). In addition, it is postulated that adding pro-angiogenic regenerative approaches could improve overall long-term limb salvage over standard of care only. To this end, several gene- and cell-based products have been tested for therapeutic angiogenesis in preclinical animal models and clinical trials. Among gene therapies, previously studied constructs have largely been formulated as naked DNA plasmids encoding soluble angiogenic factors, notably vascular endothelial growth factor (VEGF) (4–6), hepatocyte growth factor (HGF) (7, 8), and fibroblast growth factor (FGF) (9–11). These therapies have shown promise for improving hemodynamic parameters and ulcer healing in CLTI, but without clear benefit on major amputation rates, amputation-free survival, or overall survival in randomized controlled studies (12).

To address the treatment gap in CLTI, we have previously studied the cell adhesion molecule (CAM), E-selectin, as a novel target for therapeutic angiogenesis (13, 14). E-selectin is a multifunctional CAM expressed by activated endothelium that mediates rolling and extravasation of neutrophils and monocytes, thereby playing an important role in inflammatory, immunologic, and thrombotic processes. More recently, the pro-angiogenic actions of E-selectin signaling have also been elucidated. In response to tissue injury, release of cytokines/chemokines, including stromal cell-derived factor 1α (SDF-1α), induces local endothelial expression of membrane-bound E-selectin and systemic reciprocal E-selectin ligand expression on endothelial progenitor cells (EPCs) in the bone marrow *via* C-X-C motif chemokine receptor 4 (CXCR4) (15). Thus, SDF-1α initiates an E-selectin/E-selectin ligand signaling cascade that mobilizes bone marrow-derived EPCs to areas of wound healing and ischemia where these cells can then contribute to vasculogenesis and neovascularization (16). Our previous work in murine hindlimb ischemia models showed that local overexpression of E-selectin by intramuscular gene transfer can be used to make the tissue microenvironment more receptive to EPCs, potentiating the endogenous angiogenic response with corresponding benefits on wound healing (13) and footpad tissue loss (14). This focus on modification of cell-bound adhesion *via* E-selectin is the major novelty introduced by our work. In contrast to most clinical trials of gene-based therapeutic angiogenesis, our prior studies of E-selectin gene therapy used an adeno-associated virus (AAV) delivery vector to provide higher transduction efficiency than naked plasmids with lower immunogenicity than adenoviral vectors (17). Several AAV serotypes exist with diverse tissue tropisms based on capsid- and tissue-specific differences in cellular uptake and intracellular trafficking (18). In our previous work, we employed serotype AAV2/9 due to its known tropism for muscle, both cardiac and skeletal (19). On the other hand, serotype AAV2/2 has been the most widely studied with broad tissue tropism toward skeletal muscle, neurons, vascular smooth muscle cells, and hepatocytes, as well as favorable *in vitro* transduction efficiency (20). Intramuscular AAV2/2 therapies have been used in Phase I studies to treat hemophilia B and α_1_-antitrypsin deficiency (21, 22), and both AAV2/2 and AAV2/9-based gene therapies are currently approved by the United States Food and Drug Administration.

As such, the goals of this study were to evaluate the efficacy of E-selectin/AAV2/2 (E-sel/AAV2/2), a novel gene construct for therapeutic angiogenesis, for improving limb salvage, reperfusion, and function in a clinically relevant murine gangrene model. Additionally, we sought to characterize the temporal expression of E-selectin in response to ischemia, both in treated and untreated muscle, as well as the downstream angiogenic and inflammatory responses induced by E-sel/AAV2/2 gene therapy.

## Materials and Methods

### Production of Adeno-Associated Vectors

Murine *E-selectin* and *LacZ* genes were inserted into multiple cloning sites in the pZac vector (13). After confirmation by gene sequencing, E-selectin/pZac and LacZ/pZac plasmids were sent to the University of North Carolina Gene Therapy Vector Core where AAV serotype 2/2 was prepared by three-plasmid transfection into HEK293 cells (23). Quality assurance and control testing was performed by PCR quantification of genomes and infectivity titer.

### Administration of E-sel/AAV2/2 Gene Therapy

All animal experiments described were approved by the University of Miami Institutional Animal Care and Use Committee under protocol 19-163. To account for the lag time between injections and tissue transgene expression, gene therapy was administered 4 and 2 days prior to surgery, as well as on the day of left femoral artery and vein coagulation. Total dose was 1 × 10^11^ viral genome divided across the 3 days and diluted in 100 μL phosphate-buffered saline (PBS) per dose per mouse. While anesthetized with inhaled isoflurane 1.5–2% and oxygen at 2 L/min, E-sel/AAV2/2 (*N* = 31, 17 female) and LacZ/AAV2/2 (*N* = 23, 12 female) were administered with a 31-gauge needle *via* intramuscular (IM) injections of 20 μL each into the left adductor group (2), lateral thigh (1), and medial (1) and lateral (1) gastrocnemius.

### Induction of Hindlimb Gangrene

FVB/NJ male and female mice (001800, Jackson Laboratory, Bar Harbor, ME) aged 10–12 weeks were anesthetized by intraperitoneal (IP) injection of 80 mg/kg ketamine and 5 mg/kg xylazine diluted in PBS. Hair was removed from bilateral groins and hindlimbs and the left groin was prepped with chlorohexidine. A 1 cm incision was made in the left groin and the subcutaneous fat dissected laterally off the inguinal ligament. The femoral sheath was pierced with fine forceps and the femoral nerve gently dissected from the femoral vessels. After identifying the lateral circumflex femoral artery (LCFA), an electrocautery device was used to coagulate and transect both the femoral artery and vein just proximal to the LCFA. In similar fashion, the femoral vessels were divided more distally between the superficial caudal epigastric artery and the saphenopopliteal bifurcation, taking care not to injure the femoral nerve. After ensuring hemostasis, the wound was closed with 5-0 absorbable suture. To further increase tissue oxidative stress, *N*_ω_-Nitro-L-arginine methyl ester hydrochloride (L-NAME) (N5751, Sigma-Aldrich, St. Louis, MO), a competitive inhibitor of nitric oxide synthase, was dissolved in PBS and administered 2 h preoperatively and on postoperative days (POD) 1–3 *via* IP injection (40 mg/kg per injection).

### Laser Doppler Perfusion Imaging

Hindlimb perfusion was assessed using a moorLDI laser Doppler perfusion imaging (LDPI) device running version 5 software (Moor Instruments, Wilmington, DE). Mice were anesthetized with inhaled isoflurane 1.5–2% and oxygen at 2 L/min and placed in prone position with the extremities taped in place on a black foam mat with a temperature-controlled heating pad. Perfusion index was calculated as the ratio of mean flux values from the left/ischemic relative to right/non-ischemic hindlimb.

### Faber Hindlimb Ischemia Scoring

Footpad tissue loss was evaluated by Faber hindlimb ischemia scoring on POD 1–3, 7, 14, and 21. Faber scores range from 0 to 12 with 0 to 5 corresponding to the number of ischemic nails, 6–10 corresponding to 1–5 ischemic digits, and 11–12 reflecting partial or complete foot atrophy (24). Functionally, there is an important distinction between ischemia of nails compared to digits, so we defined severe gangrene as Faber score >5 and evaluated presence or absence of severe gangrene as a separate outcome.

### Treadmill Exhaustion Testing

Mice (*N* = 9 per group) were trained to run on an Exer 3/6 treadmill (Columbus Instruments, Columbus, OH) during four separate sessions spanning over 2 weeks preoperatively. For training sessions, mice walked on the treadmill with a 10° incline at a speed of 10 m/min for 10 min and then 15 m/min for 5 min with shocks enabled at 1 Hz. After the training period, mice were randomly separated into two groups. For treadmill exhaustion testing, mice were placed on the treadmill and allowed to acclimate with the belt off and shocks on for 5 min. The treadmill was then started at a speed of 5 m/min at a 10° incline and speed was ramped up by 1 m/min^2^. Distance recording started when speed reached 10 m/min, after which speed was further increased to 15 m/min after 5 min, and then incrementally increased by 3 m/min every 5 min thereafter until maximum speed of 30 m/min. Exhaustion was defined as 40 shocks, at which point shocks were disabled and total walking distance was recorded. Treadmill exhaustion testing was performed on POD 7, 10, 14, and 21.

### DiI Perfusion and Confocal Laser Scanning Microscopy

To assess neovascularization in calf muscle, intracardiac perfusion of the lipophilic carbocyanine dye 1,1'-dioctadecyl-3,3,3',3'-tetramethylindocarbocyanine perchlorate (DiI) was performed on POD 22 (*N* = 6 per group). DiI solution (D282, Invitrogen/Thermo Fisher Scientific, Waltham, MA), diluent, and fixative were prepared, and the perfusion apparatus was assembled according to previously published protocols (25–27). After euthanasia by CO_2_ overdose, the thoracic cavity was exposed, and the right atrial appendage incised while inserting the 25-gauge butterfly needle of the perfusion apparatus into the left ventricle. Sequentially, 4 mL of PBS, 10 mL of DiI, and 10 mL of 10% neutral-buffered formalin were injected after which the left and right calf muscles were harvested. Tissues were kept in fixative overnight before proceeding to imaging. To mount tissues, the entire calf muscle was sandwiched between two glass microscope slides and compressed using small binder clips. A Leica TCS SP5 (Leica Microsystems, Wetzlar, Germany) inverted confocal microscope was used to image tissues under 5X magnification. To control for technical errors during perfusion, we imaged bilateral gastrocnemius muscles and calculated the ratio of vessel density between left/ischemic and right/non-ischemic hindlimbs. Quantification of tissue vascularity was performed in Fiji (National Institutes of Health, Bethesda, MD).

### Histological Examination

On POD 21, mice underwent euthanasia and intracardiac perfusion with 10% neutral-buffered formalin prior to harvesting the left and right adductor and calf muscles. Tissue samples were embedded in paraffin and sectioned. Slides were deparaffinized, hydrated, washed, and stained with hematoxylin (95057-844, VWR, Radnor, PA) and eosin (95057-848, VWR). Slides were imaged with a Leica DFC295 (Leica Microsystems) under 20X and 40X magnification. To measure myofiber cross-sectional area, 2 sections per animal (*N* = 5 per group) were imaged under 20X magnification with a Zeiss Axio Observer inverted microscope (ZEISS, Oberkochen, Germany). Four muscle fiber perimeters were traced per image and the cross-sectional area was calculated in ZEN software (version 3.3, ZEISS). Results are expressed both in terms of absolute myofiber cross-sectional area and relative to that of non-ischemic muscle.

### Immunofluorescence Assays

Immunofluorescence studies were performed in calf muscle harvested on POD 21 to evaluate for expression of E-selectin and presence of EPCs and inflammatory cells. Slides were deparaffinized per standard protocol and antigen retrieval was performed in EDTA buffer (pH 9.0) at 120°C for 10 min. Slides were then washed in distilled water and permeabilized with 0.25% Triton-X100 TBS for 15 min. Tissue was incubated with Protein Block (ab64226, Abcam, Cambridge, United Kingdom) for 1 h. Slides were then incubated overnight at 4°C with conjugated primary antibodies, including E-selectin/CD62E Alexa Fluor 488 (5 μg/mL) (NB110-85473AF488, Novus Biologicals, Littleton, CO), CD31/PECAM-1 Alexa Fluor 647 (5 μg/mL) (NB600-1475AF647, Novus Biologicals), KDR/VEGFR-2 Alexa Fluor 647 (5 μg/mL) (NBP1-43300AF647, Novus Biologicals), CD34 AF488 (5 μg/mL) (SC-18917AF488, Santa Cruz Biotechnology, Dallas, TX) CD3 FITC (5 μg/mL) (11-0032-82, eBioscience/Thermo Fisher Scientific), and unconjugated Mac-2/Galectin-3 (5 μg/mL) (125401, BioLegend, San Diego, CA) followed by Alexa Fluor 594 goat anti-rat IgG (2 μg/mL) (A11007, Invitrogen). To confirm antibody specificity, isotype-matched negative controls were performed using rat IgG2a, kappa monoclonal antibody (ab18450, Abcam) (5 μg/mL) with Alexa Fluor 594 goat anti-rat IgG (2 μg/mL) as secondary antibody (Supplementary Figure 1). Slides were imaged with an Axio Observer inverted microscope (ZEISS). For each stain, 4 images were acquired at 20X magnification from 4 sections per animal (*N* = 5 per group) and cell counting or quantification of mean fluorescence intensity was performed in Fiji.

### RT-qPCR, PCR Arrays, and Bioinformatics Analysis

Thigh and calf muscles were harvested at specified time points postoperatively and total RNA was isolated after tissue homogenization in TRIzol reagent (15596018, Invitrogen/Thermo Fisher Scientific) according to manufacturer instructions. Total RNA was reverse transcribed using RT^2^ First Strand Kit (Qiagen, Venlo, Netherlands). Real-time reverse transcription quantitative polymerase chain reaction (RT-qPCR) was performed using RT^2^ SYBR Green qPCR Mastermix (330500, Qiagen) and primers for *E-sel* (*Sele*, NM_0113435, assay ID Mm.PT.58.11296882) and *Rplp0* (NM_007475, assay ID Mm.PT.58.43894205) as housekeeping gene (Integrated DNA Technologies, Coralville, IA). PCR arrays assessing 84 genes related to angiogenesis (RT^2^ Profiler PCR Array Mouse Angiogenesis, GeneGlobe ID PAMM-024Z, Qiagen) and inflammation (RT^2^ Profiler PCR Array Mouse Inflammatory Cytokines & Receptors, GeneGlobe ID PAMM-011Z, Qiagen) were performed according to manufacturer instructions using a Bio-Rad CFX96 Touch Real-Time PCR Detection System (Bio-Rad Laboratories, Hercules, CA). Data were analyzed using the ΔCt method (2^−Δ*ΔCt*^) method and RT-qPCR and PCR arrays were performed in duplicate using samples from 3 to 6 mice per group. For bioinformatics analysis, raw expression (Ct) values were normalized to housekeeping genes (*Actb, B2m, Gapdh, Gusb, Hsp90ab1*) using R Statistical Software (R Core Team 2021) “NormqPCR” (28). Then, -ΔCt values were analyzed for differential expression using R “limma” (29), and raw *P*-values based on empirical Bayes moderated *t*-statistics were adjusted for multiple testing with Benjamini & Hochberg false discovery rate correction (30).

### Evaluation of Coagulation and Hematologic, Hepatic, and Renal Function

Intracardiac blood samples collected on POD 21 were sent for complete blood count and comprehensive metabolic panel. To evaluate for prothrombotic effect of E-selectin/AAV2/2, blood samples were collected on POD 7 in tubes containing 3.2% sodium bicarbonate in 9:1 ratio and centrifuged at 3,000 rpm for 10 min. The supernatant plasma was collected and used for enzyme-linked immunosorbent assay (ELISA) quantification of D-dimer levels per the kit protocol (LS-F6179-1, LifeSpan BioSciences, Seattle, WA) using a previously determined optimal sample dilution of 1:1,000.

### Statistics

Statistical analyses were performed using GraphPad Prism (version 9.0.1, GraphPad Software, San Diego, CA). All continuous data were normally distributed according to Shapiro-Wilk test and compared using Student's *t*-test for two variables and ANOVA for greater than two variables. Categorical outcomes between two groups were compared using Chi-square test. Data are presented as mean ± standard error (SEM) and results with *P* < 0.05 considered statistically significant.

## Results

### E-sel/AAV2/2 Gene Therapy Provides High-Level and Durable Tissue Transgene Expression

**Figure 1:**
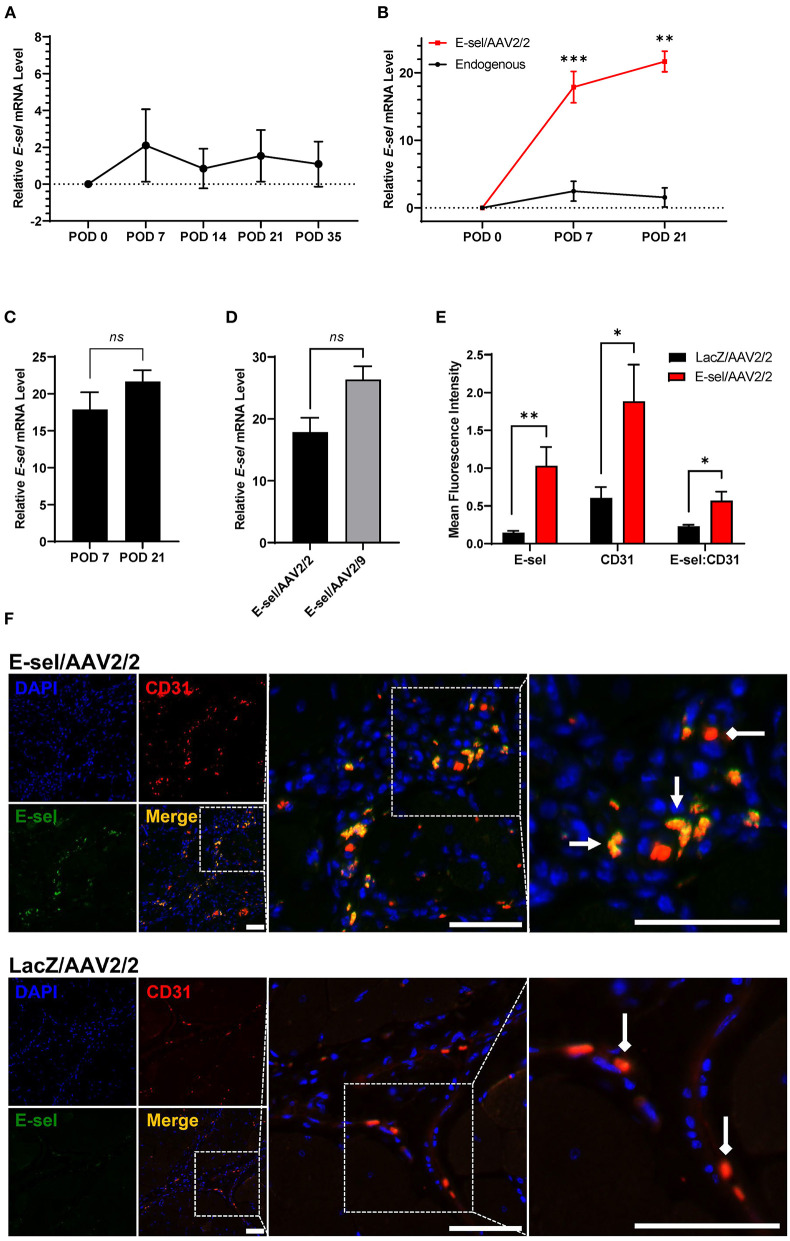
High-level E-selectin expression by E-sel/AAV2/2 treatment in ischemic limb tissues. **(A)** Endogenous *E-sel* mRNA measured by RT-qPCR is transiently upregulated in untreated ischemic muscle normalized to untreated non-ischemic muscle. **(B)** Relative *E-sel* mRNA in E-sel/AAV2/2-treated ischemic muscle normalized to LacZ/AAV2/2-treated ischemic muscle (red) demonstrates high-level *E-sel* expression compared to endogenous levels in untreated ischemic muscle (black). **(C)** *E-sel* mRNA levels are stable from 1 to 3 weeks after treatment with E-sel/AAV2/2. **(D)** Comparison of *E-sel* mRNA expression by AAV2/2 and AAV2/9 vectors demonstrating no significant difference on POD 7. **(E)** Quantification and **(F)** representative images of immunofluorescence staining for CD31 (red) and E-selectin (green) showing enhanced colocalization (yellow) of these markers in muscle treated with E-sel/AAV2/2 (pointed arrowheads) compared to CD31 staining in absence of E-selectin positivity in muscle treated with LacZ/AAV2/2 (diamond arrowheads). Scale bars represent 50 μm. Data are presented as mean ± SEM where *P < 0.05, **P < 0.01, ***P < 0.001, and *ns*, not significant (*P* > 0.05).

To account for the lag time between AAV injection and tissue transgene expression, our protocol called for gene therapy administration 4 and 2 days preoperatively, and immediately prior to induction of hindlimb ischemia. A total of 1 × 10^11^ viral genome divided across 3 doses of either E-sel/AAV2/2 or LacZ/AAV2/2 serving as control was administered to 5 sites in the left thigh adductor and calf muscles. Expression of transgene, *E-selectin* mRNA (*E-sel*), in hindlimb tissues was measured by RT-qPCR. Relative to non-ischemic hindlimb, endogenous *E-sel* levels in untreated ischemic muscle at various time points after femoral artery and vein coagulation were 2.10 ± 1.97 on POD 7, 0.84 ± 1.08 on POD 14, 1.54 ± 1.40 on POD 21, and 1.09 ± 1.22 on POD 35 (Figure 1A). While there was a trend toward increased *E-sel* levels above baseline on POD 7, this result was not statistically significant (*P* = 0.085). Moreover, any endogenous elevation in tissue *E-sel* was transient with a rapid decline to baseline levels by POD 14 which persisted through POD 35.

On the other hand, quantification of *E-sel* levels by RT-qPCR in muscle harvested from ischemic limbs treated with E-sel/AAV2/2 revealed high-level *E-sel* transgene expression by POD 7 compared to endogenous *E-sel* levels (Figure 1B). Additionally, *E-sel* levels following treatment with AAV2/2 serotype further increased from POD 7 (17.86 ± 2.331) to POD 21 (21.66 ± 1.53) although this difference was not statistically significant (*P* = 0.613) (Figure 1C). We also found comparable transduction efficiency of *E-sel* transgene expression between AAV2/2 (17.86 ± 2.33) and AAV2/9 serotypes (26.35 ± 2.14) on POD 7 (*P* = 0.299) (Figure 1D). Immunofluorescence analysis (IFA) confirmed significantly greater E-sel expression as measured by mean fluorescence intensity (MFI) in muscle treated with E-sel/AAV2/2 compared to LacZ/AAV2/2 (1.031 ± 0.249 vs. 0.144 ± 0.025, *P* = 0.008; Figures 1E,F). Thus, both assays used to detect E-sel gene expression and protein levels demonstrated that intramuscularly administered E-sel/AAV2/2 provides high-level and durable tissue transgene expression in ischemic limb muscle. Additionally, on IFA, E-sel/AAV2/2 gene therapy was associated with increased capillary density as measured by CD31 MFI (1.884 ± 0.486 vs. 0.605 ± 0.144, *P* = 0.036) and an elevated E-selectin to CD31 MFI ratio (0.569 ± 0.121 vs. 0.229 ± 0.021, *P* = 0.024; Figure 1E), suggesting a pro-angiogenic effect of E-sel/AAV2/2 gene therapy that is consistent with further observations in this study.

### E-sel/AAV2/2 Gene Therapy Promotes Pro-angiogenic Response and Increases Footpad Reperfusion and Calf Muscle Vascularity

**Figure 2:**
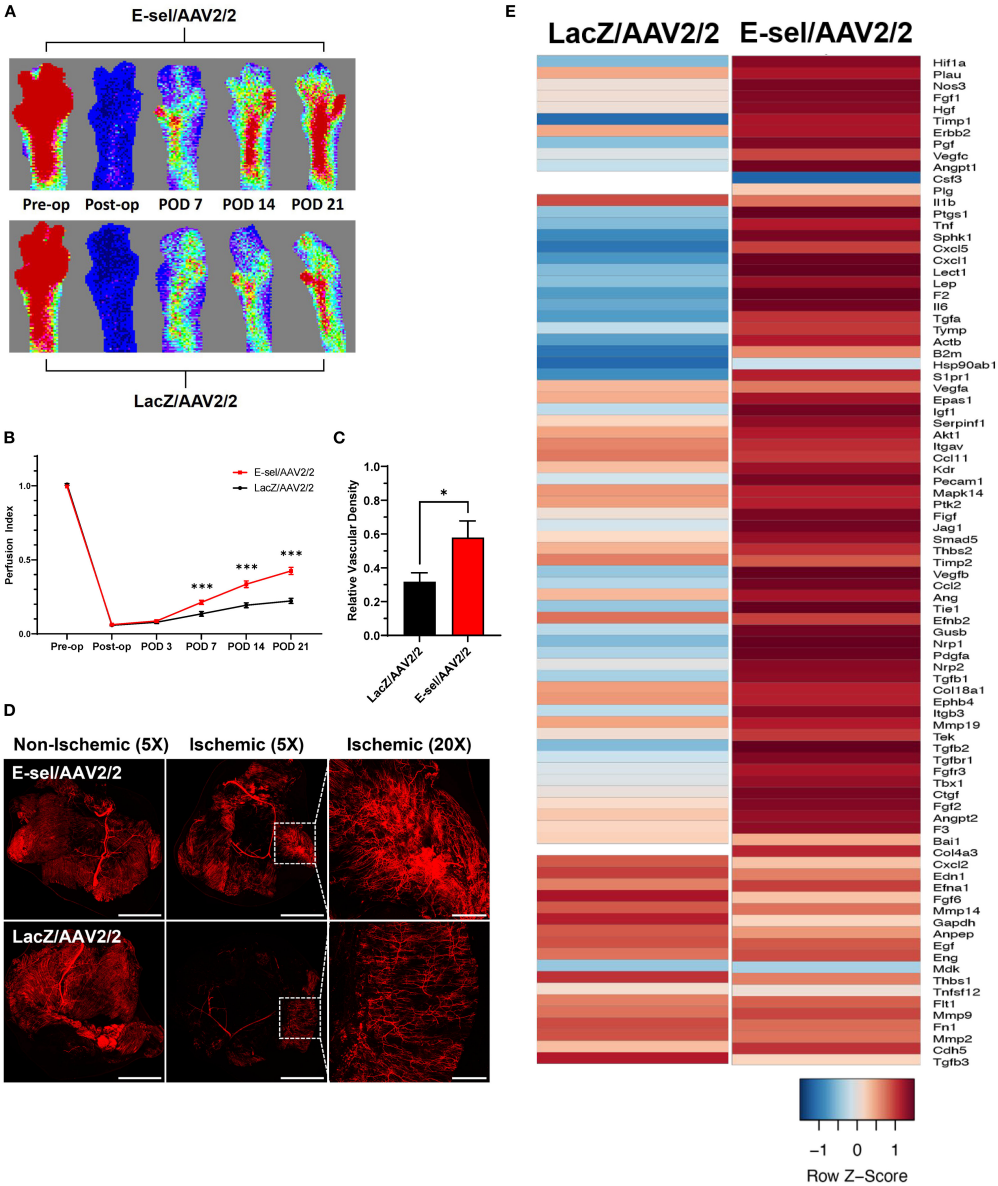
Hindlimb reperfusion and angiogenesis gene profile is enhanced by E-sel/AAV2/2. **(A)** Representative laser Doppler perfusion images and **(B)** quantification of perfusion indices demonstrating improved recovery of footpad perfusion in mice treated with E-selectin/AAV2/ compared to LacZ/AAV2/2. **(C)** Quantification and **(D)** representative confocal microscopy images of calf muscle following whole-body perfusion with DiI to stain the peripheral vasculature on POD 22. Scale bars represent 5 and 1 mm for 5X and 20X images, respectively. **(E)** PCR array demonstrating upregulation of several angiogenesis genes in muscle treated with E-sel/AAV2/2 compared to LacZ/AAV2/2 control vector. Data are presented as mean ± SEM where *P < 0.05 and ***P < 0.001.

To measure the angiogenic effect of E-sel/AAV2/2 gene therapy, we quantified ischemic limb reperfusion on LDPI and visualized skeletal muscle vascularity by whole-body DiI perfusion and subsequent confocal microscopy of resected ischemic calf muscles. On LDPI, a significant improvement in footpad perfusion index, expressed as the ratio of blood flow of the ischemic relative to non-ischemic limb, was observed with E-sel/AAV2/2 therapy compared to LacZ/AAV2/2 control starting on POD 7 (0.213 ± 0.014 vs. 0.135 ± 0.016, *P* < 0.001) with a continued steady increase through the study endpoint on POD 21 (0.425 ± 0.024 vs. 0.222 ± 0.017, *P* < 0.001; Figures 2A,B). Because LDPI measurements primarily reflect blood flow *via* larger caliber arteries, we also examined small blood vessel and capillary density in the treated limb tissues by whole-body DiI perfusion and subsequent laser scanning confocal microscopy of resected calf muscles which allows for high-resolution, three-dimensional visualization of tissue vasculature (27). Imaging of ischemic gastrocnemius muscle after DiI perfusion on POD 22 showed that mean vessel density was significantly greater in mice treated with E-sel/AAV2/2 (0.579 ± 0.099) compared to LacZ/AAV2/2 controls (0.317 ± 0.054) (*P* = 0.042; Figures 2C,D), further providing evidence that E-sel/AAV2/2 gene therapy is pro-angiogenic and can increase footpad reperfusion and vessel density in treated ischemic limb tissues.

To further investigate the pro-angiogenic effect of E-sel/AAV2/2 gene therapy, we carried out an angiogenesis-focused PCR array analysis to quantitatively assess 84 genes comprising a panel of growth factors, cytokines, cell adhesion molecules, and transcription factors known to play a role in angiogenesis and arteriogenesis (RT^2^ Profiler PCR Array Mouse Angiogenesis, GeneGlobe ID PAMM-024Z, Qiagen). Bioinformatic analysis was then performed using PCR array data to generate corresponding angiogenesis heatmaps (Figure 2E). The complete list of angiogenesis-related genes analyzed is available in Supplementary Figure 2. PCR array analysis demonstrated a robust angiogenic gene expression profile in ischemic muscle treated with E-sel/AAV2/2 compared to LacZ/AAV2/2 with upregulation of 14/84 genes (16.7%). Genes that were modulated more than two-fold are shown in Table 1. Taken together, these results demonstrated that E-sel/AAV2/2 gene therapy initiates a robust local angiogenic response with upregulation of diverse pro-angiogenesis genes.

**Table 1:** Name and function of angiogenesis genes modulated by intramuscular E-selectin/AAV2/2 gene therapy.

**Gene**	**Name**	**Fold-change in mRNA level**	**Function**
*Lep*	Leptin	9.48 ± 0.65	Adipokine with anti-obesity effect involved in wound healing and angiogenesis (36, 37)
*Il6*	Interleukin 6	4.24 ± 0.39	Cytokine with acute pro-angiogenic and chronic anti-angiogenic properties (35, 36)
*Tnf*	Tumor necrosis factor	3.94 ± 0.93	Cytokine involved in inflammatory angiogenesis (46, 47)
*Tymp*	Thymidine phosphorylase	3.63 ± 0.73	PD-ECGF, improves perfusion in rabbit hindlimb ischemia model (38)
*Tgfa*	Transforming growth factor α	3.31 ± 0.97	Binds EGFR, involved in post-infarct angiogenesis in the brain (48)
*S1pr1*	Sphingosine-1-phosphate receptor 1	2.90 ± 0.82	Receptor for S1P, involved in pericyte and vascular smooth muscle cell recruitment (49–53)
*Timp1*	Tissue inhibitor of metalloproteinase 1	2.61 ± 0.06	Involved in remodeling of ECM by regulating MMP activity (54)
*Pgf*	Placental growth factor	2.49 ± 0.73	Improves perfusion and exercise tolerance in rabbit hindlimb ischemia model (39)
*Sphk1*	Sphingosine kinase 1	2.27 ± 0.20	Phosphorylates sphingosine to S1P, involved in ischemic preconditioning-induced cardioprotection (55)
*Itgb3*	Integrin β3	2.23 ± 0.87	Pro-angiogenic adhesion molecule involved in endothelial cell adhesion and migration (41)
*Tbx1*	T-box 1	2.19 ± 0.94	Transcription factor required for organization and differentiation of vascular networks (56, 57)
*Ccl2*	C-C motif chemokine ligand 2	2.14 ± 0.69	MCP-1, involved in monocyte recruitment and required for arteriogenesis (40)
*Hif1a*	Hypoxia-inducible factor 1α	2.09 ± 0.81	Primary hypoxia response element leading to VEGF gradient expression (58)
*Fgfr3*	Fibroblast growth factor receptor 3	2.06 ± 0.93	Receptor for FGF, involved in vascular development (59)

*Data are presented as mean ± SD. PD-ECGF, platelet-derived endothelial cell growth factor; EGFR, epidermal growth factor receptor; S1P, sphingosine-1-phosphate; ECM, extracellular matrix; MMP, matrix metalloproteinase; MCP-1, monocyte chemoattractant protein 1; VEGF, vascular endothelial growth factor; FGF, fibroblast growth factor*.

### E-sel/AAV2/2 Gene Therapy Promotes Recruitment of EPCs to Ischemic Tissue

**Figure 3:**
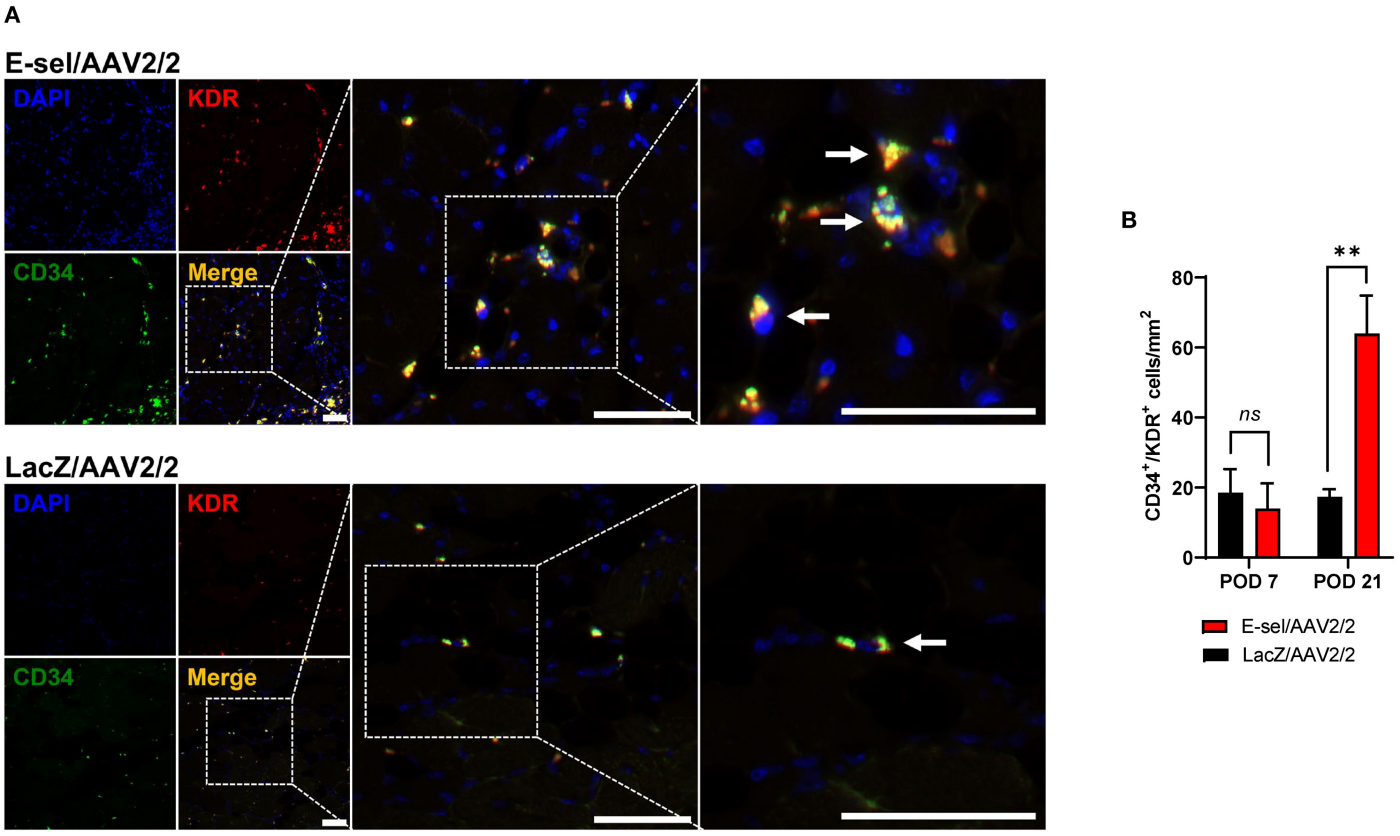
Recruitment of endothelial progenitor cells (EPCs) to ischemic muscle is enhanced by E-sel/AAV2/2. **(A)** Representative immunofluorescence images demonstrating enhanced colocalization (yellow) of EPC markers CD34 (green) and KDR/VEGFR-2 (red) on POD 21 in muscle treated with E-sel/AAV2/2 compared to LacZ/AAV2/2. EPCs indicated by pointed arrowheads. Scale bars represent 50 μm. **(B)** Counting of cells co-staining for CD34 and KDR/VEGFR-2 revealed similar EPC numbers in the acute phase of ischemia (POD 7) but enhanced recruitment of EPCs by POD 21 in muscle treated with E-sel/AAV2/2 compared to LacZ/AAV2/2. Data are presented as mean ± SEM where **P < 0.01 and *ns*, not significant (*P* > 0.05).

E-selectin has previously been demonstrated to be involved in recruitment of EPCs (15, 16). To further elucidate the mechanism underlying therapeutic angiogenesis induced by E-sel/AAV2/2 gene therapy in ischemic hindlimb, we therefore assessed recruitment of EPCs into E-sel/AAV2/2-treated vs. LacZ/AAV2/2-treated ischemic calf muscle using IFA. To identify EPCs in ischemic calf muscle, we stained for CD34 (hematopoietic cell marker carried by circulating stem/progenitor cells) and KDR (kinase insert domain receptor, also known as VEGFR-2 or vascular endothelial growth factor receptor 2, an endothelial cell marker; Figure 3A). In limb tissues harvested on POD 7, we found no significant difference in EPC numbers in muscle treated with E-sel/AAV2/2 compared to LacZ/AAV2/2 (14.0 ± 7.2 cells/mm^2^ vs. 18.5 ± 6.7 cells/mm^2^, *P* = 0.670). By POD 21, however, IFA demonstrated significantly more CD34^+^/KDR^+^ colocalization in ischemic tissue treated with E-sel/AAV2/2 compared to LacZ/AAV2/2 (63.9 ± 10.9 cells/mm^2^ vs. 17.3 ± 2.2 cells/mm^2^, *P* = 0.003), indicating enhanced EPC recruitment in the treatment group (Figure 3B). Globally, ischemic tissue from LacZ/AAV2/2-treated mice demonstrated sparse foci of EPC recruitment, whereas in E-sel/AAV2/2-treated limb muscle, there were consistently several large foci of EPC enrichment. We found no appreciable numbers of CD34^+^/KDR^+^ cells in non-ischemic limb muscle from either group. The data, hence, suggested that E-sel/AAV2/2-induced therapeutic angiogenesis is mediated, at least in part, by increasing recruitment of circulating EPCs to ischemic tissues where these cells can then participate in neovascularization.

### E-sel/AAV2/2 Gene Therapy Reduces Severity of Tissue Loss and Incidence of Severe Gangrene

**Figure 4:**
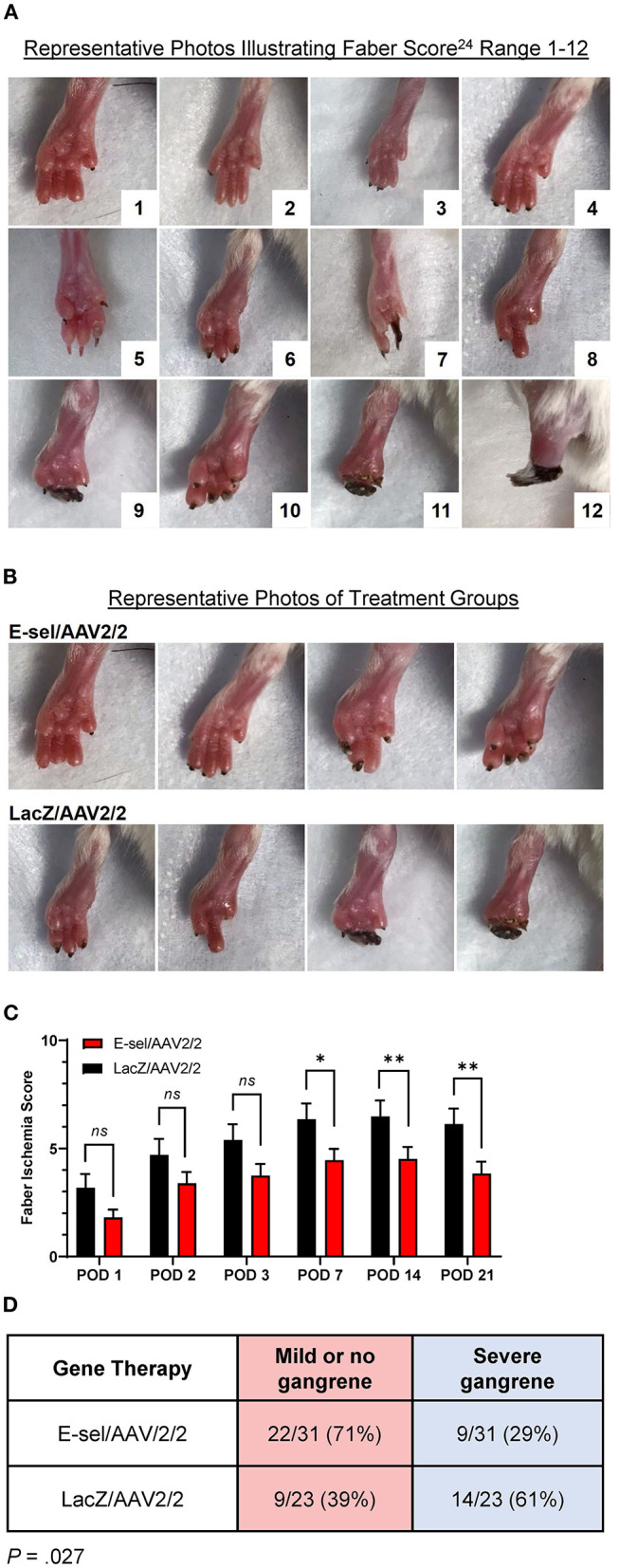
Limb tissue loss and incidence of severe gangrene is reduced by E-sel/AAV2/2 gene therapy. **(A)** Representative photos illustrating range of Faber ischemia scores 1–12. **(B)** Representative images of footpads obtained on POD 14 in mice treated with E-sel/AAV2/2 and LacZ/AAV2/2. **(C)** Mean Faber ischemia scores up to POD 21 after induction of hindlimb ischemia are significantly lower in mice treated with E-sel/AAV2/2 compared to LacZ/AAV2/2. **(D)** Proportion of mice with severe gangrene (Faber score >5) on POD 14. Data are presented as mean ± SEM where *P < 0.05, **P < 0.01, and *ns*, not significant (*P* > 0.05).

To evaluate the therapeutic effect of E-sel/AAV2/2 gene therapy on severity of tissue loss, we monitored and recorded Faber scores at prespecified time points. The Faber hindlimb ischemia score is an established and popular grading scheme used to assess severity of tissue loss in hindlimb ischemia models (24). Examples of Faber scores 1–12 are shown in Figure 4A. A Faber score of 0 reflects a normal foot without any ischemic nails or digits. Mice treated with E-sel/AAV2/2 or LacZ/AAV2/2 were visually inspected on POD 1–3 and weekly through POD 21. Mean Faber scores were consistently lower for E-sel/AAV2/2-treated mice compared to LacZ/AAV2/2 controls starting on POD 7 (4.45 ± 0.53 vs. 6.35 ± 0.74, *P* = 0.042) and through POD 21 (3.84 ± 0.55 vs. 6.13 ± 0.71, *P* = 0.014; Figures 4B,C), indicating overall reduced tissue loss severity in the treatment group. Because of the significant functional difference between ischemic nails (Faber score 1–5) and ischemic/necrotic digits (Faber score 6–12), we further categorized limb outcomes as mild and severe gangrene according to these cutoff values. By POD 21, incidence of severe gangrene, defined as Faber score greater than 5, was 29% (9/31) in the E-sel/AAV2/2 group compared to 61% (14/23) in the LacZ/AAV2/2 group (*P* = 0.027), corresponding to an absolute risk reduction of 31.8% and relative risk of 0.478 (95% confidence interval 0.248–0.889; Figure 4D). Overall, these data quantifying gross limb appearance showed that E-sel/AAV2/2 gene therapy reduces severity of limb tissue loss and incidence of severe gangrene, demonstrating its efficacy for limb salvage.

### E-Sel/AAV2/2 Gene Therapy Preserves Histological Integrity and Improves Function of Ischemic Muscle

**Figure 5:**
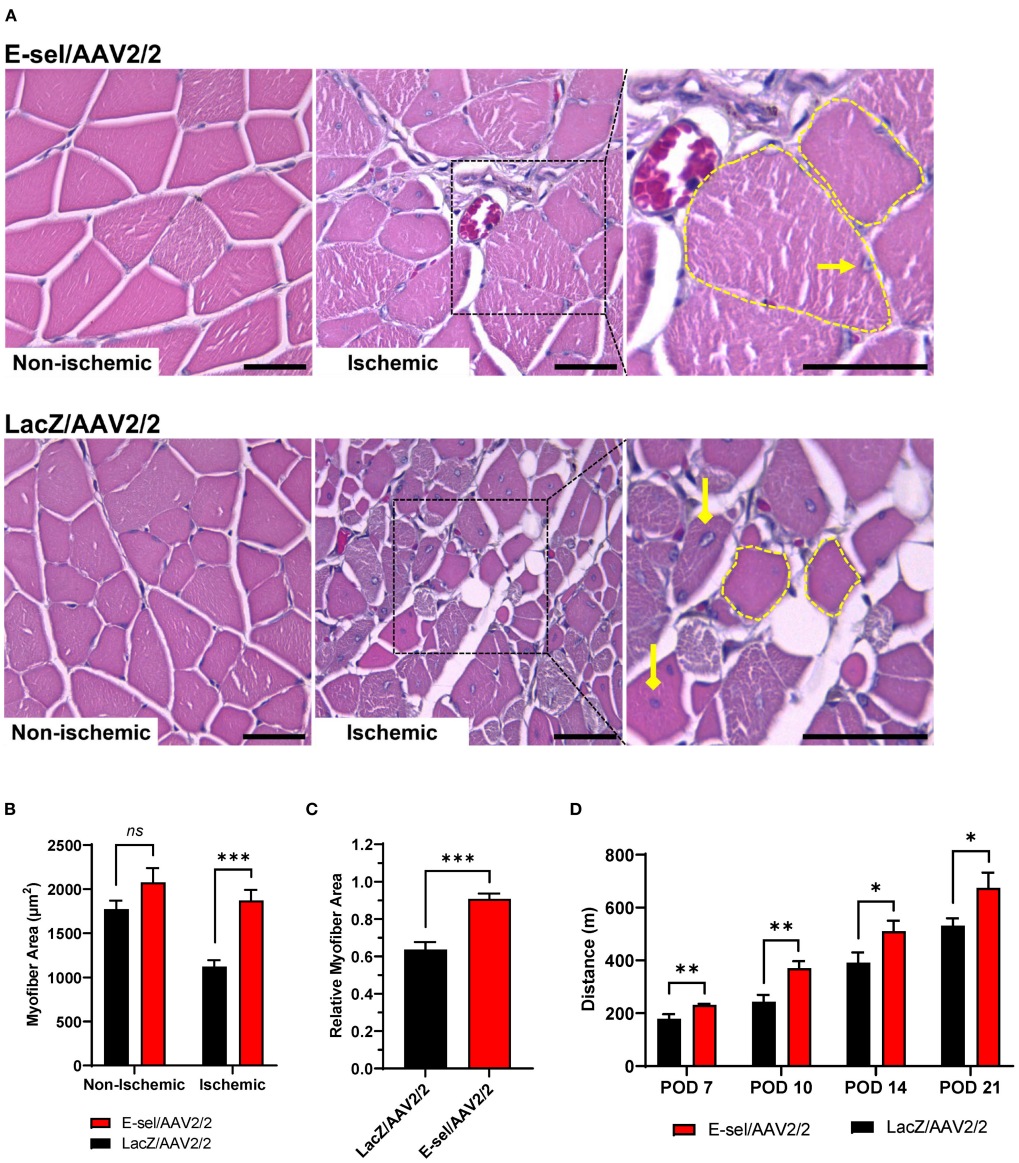
E-sel/AAV2/2 gene therapy is associated with preserved myofiber integrity and improved functional recovery in ischemic hindlimb. **(A)** Representative H&E sections demonstrating better-preserved muscle integrity with larger myofiber size (dashed yellow line) and peripherally located nuclei (pointed arrowhead) in E-sel/AAV2/2-treated muscle compared to shrunken (dashed yellow line), eosinophilic necrotic fibers and centrally located nuclei (diamond arrowheads) observed in muscle treated with LacZ/AAV2/2. Scale bars represent 25 μm. **(B)** Measurement of myofiber cross-sectional area demonstrating reduced myofiber size in LacZ/AAV2/2-treated compared to E-sel/AAV2/2-treated ischemic muscle. Non-ischemic myofiber size is comparable across groups. **(C)** Relative myofiber size in ischemic muscle compared to non-ischemic muscle is significantly larger after treatment with E-sel/AAV2/2 compared to LacZ/AAV2/2. **(D)** Mean distance walked on treadmill exhaustion testing at various timepoints postoperatively. Data are presented as mean ± SEM where *P < 0.05, **P < 0.01, ***P < 0.001, and *ns*, not significant (*P* > 0.05).

To test the effect of E-sel/AAV2/2 gene therapy on recovery of ischemic limb function, we conducted mouse treadmill exhaustion testing at prespecified postoperative time points. Preoperatively, mice were trained to run on a treadmill during 4 training sessions spread across 2 weeks. Trained mice were then randomly separated into two groups for E-sel/AAV2/2 and LacZ/AAV2/2 treatment, respectively. Following induction of hindlimb gangrene, both groups suffered significant reduction in exercise capacity. Walking/running distance on treadmill exhaustion testing then progressively improved for both groups but starting on POD 7 (231 ± 4 vs. 179 ± 17 m, *P* = 0.001) and through POD 21 (675 ± 58 vs. 532 ± 27 m, *P* = 0.043), performance was significantly better in the E-sel/AAV2/2-treated group compared to LacZ/AAV2/2-treated controls (Figure 5D). To address the pathophysiological basis of improved limb function, we performed histological analysis of calf skeletal muscle harvested on POD 21. On hematoxylin and eosin (H&E) staining, myocyte integrity was better preserved in the E-sel/AAV2/2-treated group compared to LacZ/AAV2/2-treated controls, as demonstrated by increased absolute myofiber size (1,872 ± 119 vs. 1,121 ± 75 μm, *P* < 0.001) and that relative to non-ischemic calf muscle (0.907 ± 0.029 vs. 0.637 ± 0.040, *P* < 0.001; Figures 5A–C). Myofiber size in non-ischemic muscle was not significantly different between groups (2,078 ± 162 vs. 1,772 ± 97 μm, *P* = 0.145; Figure 5B). Our data thus revealed that E-sel/AAV2/2 gene therapy improves functional recovery of ischemia-injured limbs and gangrenous footpads by reducing skeletal muscle atrophy.

### Effects of E-sel/AAV2/2 Gene Therapy on Inflammation, Coagulation, and Hematologic, Hepatic, and Renal Function

**Figure 6:**
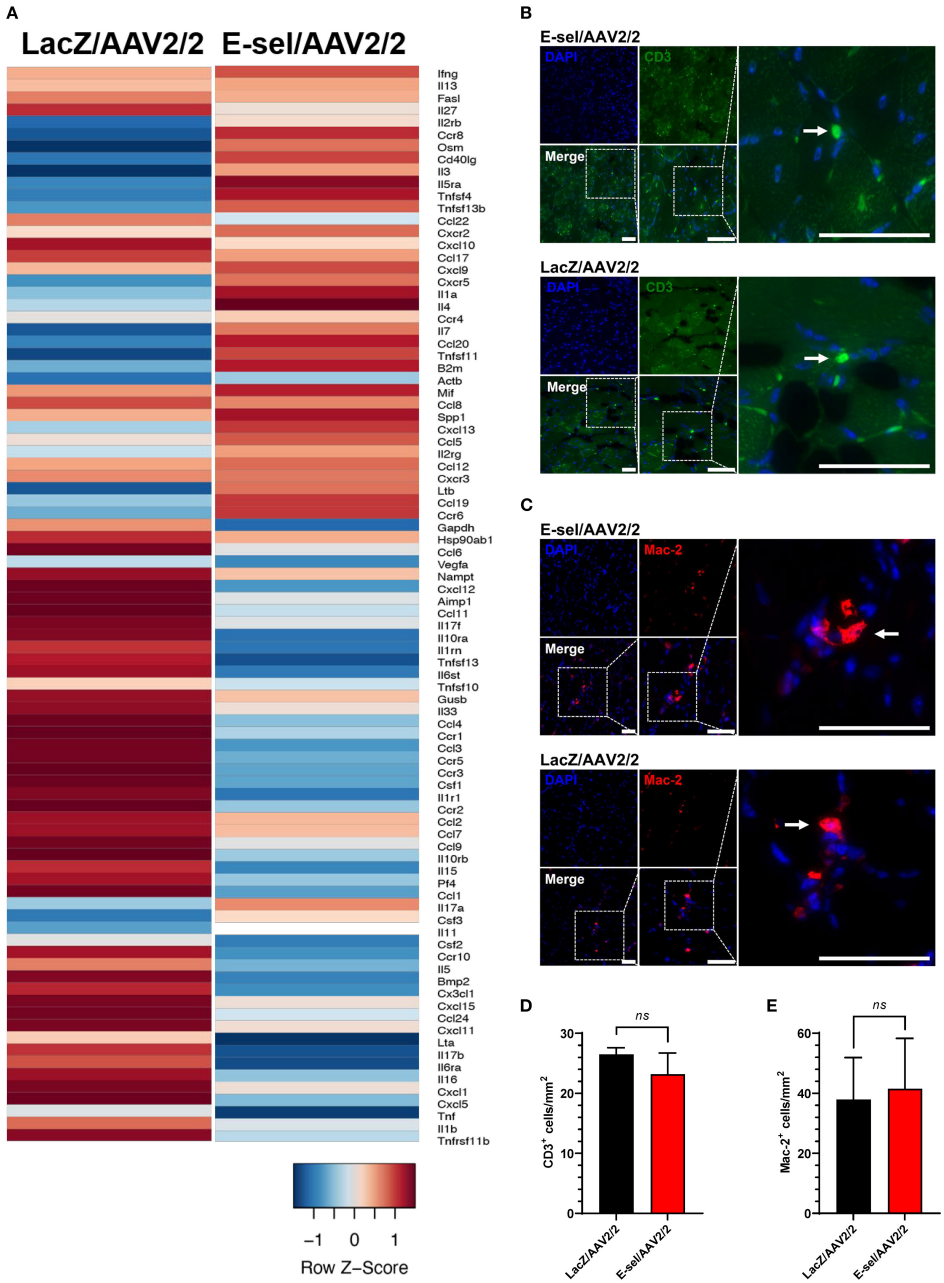
E-sel/AAV2/2 modulates inflammatory gene profile in ischemic muscle without inducing significant inflammatory cell recruitment. **(A)** PCR array from muscle harvested on POD 21 demonstrating an overall downregulation of inflammatory genes by E-sel/AAV2/2 gene therapy. **(B)** Representative immunofluorescence images of ischemic muscle stained for CD3, a marker of T cells (pointed arrowheads), and **(C)** Mac-2/Galectin-3, a marker of macrophages (pointed arrowheads). **(D)** Counting of CD3^+^ and **(E)** Mac-2^+^ cells demonstrating no significant difference in T cell or macrophage recruitment to ischemic muscle treated with E-sel/AAV2/2 and LacZ/AAV2/2. Scale bars represent 50 μm. Data are presented as mean ± SEM and *ns*, not significant (*P* > 0.05).

The above data indicate a pro-angiogenic role of E-selectin for therapeutic angiogenesis. Since E-selectin is also known to be involved in inflammation and thrombosis, we addressed potential effects of intramuscularly administered E-sel/AAV2/2 gene therapy on inflammation and thrombosis as well as general toxicity in our mouse hindlimb gangrene model. To assess whether E-sel/AAV2/2 gene therapy induced a pro-inflammatory response in ischemic tissue, we conducted pathway-focused PCR array analysis to specifically test levels of 84 genes related to the inflammatory response. Total RNA samples extracted from ischemic limb tissue on POD 21 were subjected to RT-qPCR array analysis (RT^2^ Profiler PCR Array Mouse Inflammatory Cytokines & Receptors, GeneGlobe ID PANZ-011Z, Qiagen). Although there was evidence of both upregulation of certain genes (21/84, 25%) and downregulation of others (27/84, 32%), there were overall more genes related with inflammation that were downregulated with E-sel/AAV2/2 therapy compared to LacZ/AAV2/2. As visualized by heatmap analysis (Figure 6A), these results indicated an overall “cooling” or dampening effect of E-sel/AAV2/2 gene therapy on tissue inflammatory gene profile. The complete list of analyzed genes related to inflammation is available in Supplementary Figure 3. Using IFA, inflammation was further assessed by counting the number of T cells (CD3^+^) and macrophages (Mac-2/Galectin-3^+^) infiltrated in treated ischemic limb tissues. In both E-sel/AAV2/2-treated and LacZ/AAV2/2-treated ischemic limb muscle, CD3^+^ cells were sparse (23 ± 4 vs. 27 ± 1 cells/mm^2^, *P* = 0.403; Figures 6B,D). Moreover, the number of Mac-2^+^ macrophages was similar between E-sel/AAV2/2-treated and LacZ/AAV2/2-treated muscle (41 ± 17 vs. 38 ± 14 cells/mm^2^, *P* = 0.877; Figures 6C,E), indicating that E-sel/AAV2/2 gene therapy did not exert a pro-inflammatory effect on POD 21.

Routine blood tests were performed to assess hematologic, hepatic, and renal function. Blood sampling on POD 21 revealed no differences in hematologic, hepatic, or renal function panels between mice treated with E-sel/AAV2/2, LacZ/AAV2/2, or PBS (Table 2), indicating that locally administered E-sel/AAV2/2 gene therapy does not appear to cause systemic toxicity. To test potential effect of E-sel/AAV2/2 gene therapy on thrombosis, we quantified serum D-dimer levels on POD 7. ELISA revealed that D-dimer was elevated above normal levels (250 ng/mL) in E-sel/AAV2/2-treated mice, but also LacZ/AAV2/2 and PBS controls. However, there was no significant difference in D-dimer levels between E-sel/AAV2/2-treated mice and those that received LacZ/AAV2/2 or PBS vehicle (Table 2). Thus, increased D-dimer levels may be related to recent surgical procedure or intramuscular injection but were not uniquely attributable to E-sel/AAV2/2 gene therapy. Collectively, our data showed that locally administered E-sel/AAV2/2 gene therapy alters the inflammatory gene profile of ischemic muscle but does not induce T cell or macrophage infiltration, thrombosis, or systemic toxicity.

**Table 2:** Serum D-dimer levels on POD 7 and complete blood count and liver and renal function tests obtained on POD 21 in mice treated with E-sel/AAV2/2, LacZ/AAV2/2, and PBS.

**Test**	**E-sel/AAV2/2**	**LacZ/AAV2/2**	**PBS**	**Normal range**	***P***
D-dimer (ng/mL)	587 ± 97	476 ± 61	635 ± 101	<250	*ns*
Hematocrit (%)	51.0 ± 3.6	51.5 ± 3.3	49.2 ± 1.0	34–50	*ns*
Hgb (g/dL)	12.0 ± 0.7	11.9 ± 0.5	11.3 ± 0.2	12.8–16.1	*ns*
WBC (x10^3^/μL)	3.5 ± 1.6	2.1 ± 0.7	3.5 ± 4.6	4.5–9.1	*ns*
Platelets (x10^3^/μL)	939 ± 64	989 ± 56	1,009 ± 267	100–250	*ns*
BUN (mg/dL)	20 ± 4	16 ± 1	21 ± 3	18–29	*ns*
Creatinine (mg/dL)	0.1 ± 0.1	0.1 ± 0	0.1 ± 0	0.1–0.4	*ns*
CPK (U/L)	37 ± 6	32 ± 5	50 ± 9	50–114	*ns*
Protein, total (g/dL)	5.1 ± 0.4	5.3 ± 0.6	4.8 ± 0.2	4.6–6.9	*ns*
Albumin (g/dL)	2.5 ± 0.3	2.5 ± 0.3	2.4 ± 0.2	2.5–4.8	*ns*
Bilirubin, total (mg/dL)	0.3 ± 0.2	0.3 ± 0.2	0.5 ± 0.2	0.1–0.9	*ns*
AST (U/L)	64 ± 17	75 ± 18	58 ± 3	50–270	*ns*
ALT (U/L)	39 ± 1	34 ± 7	34 ± 2	29–77	*ns*
ALP (U/L)	94 ± 7	96 ± 9	78 ± 7	51–285	*ns*

*PBS, phosphate-buffered saline; Hgb, hemoglobin; WBC, white blood cell count; BUN, blood urea nitrogen; CPK, creatine phosphokinase; AST, aspartate transaminase; ALT, alanine transaminase; ALP, alkaline phosphatase. Data are presented as mean ± SD where ns, not significant (P > 0.05)*.

## Discussion

Our prior work on E-selectin vascular regenerative approaches indicated a beneficial effect on murine limb revascularization. In this study, we demonstrate for the first time that *in vivo* direct E-sel/AAV2/2 gene therapy results in dramatic changes of the tissue angiogenesis and inflammatory gene profiles. These major findings are associated with more effective recruitment of EPCs to the compromised ischemic limb. In addition, the phenotype of the treated animals indicates significantly improved recovery in laser Doppler perfusion and capillary density with associated reduction in tissue loss. The absolute risk reduction provided by E-sel/AAV2/2 gene therapy regarding development of severe gangrene, which we defined as digital or foot necrosis (Faber score >5), was 32%. This effect size suggests a number needed to treat on the order of 3–4. While these results are not directly translatable to human subjects, the observed reduction in mean tissue loss severity and overall incidence of severe gangrene in this study demonstrates promise for local E-selectin-based gene therapy as a potential treatment for limb-threatening ischemia.

PAD/CLTI is a chronic disease in which buildup of atherosclerotic plaque typically occurs over many years. This gradual occlusive process allows for adaptation of skeletal muscle to tissue hypoxia, and how well individuals can compensate explains some of the variability in symptoms experienced by patients with PAD/CLTI. In this chronic setting, limb tissue may become more tolerant to tissue hypoxia and respond differently than it does to an acute ischemic insult. While our findings corroborate prior studies indicating that E-selectin is acutely upregulated following induction of ischemia, expression of E-selectin in chronically ischemic tissue is not well-characterized. Like others, we found that endogenous E-selectin levels rapidly return to baseline within 7 days and remain at this level afterwards (31). Such a transient and modest elevation of E-selectin is an acute response to tissue ischemia and likely insufficient to initiate or support an adequate angiogenic response essential for rescuing and repairing chronically ischemia-injured tissue. Therefore, increasing tissue levels of E-selectin and prolonging its expression, particularly in the vasculature, can aid in mounting a therapeutic angiogenic response.

The present construct, E-sel/AAV2/2, achieved stable transgene expression 20-fold higher than LacZ/AAV2/2 control vector up to 3 weeks after therapy. IFA then confirmed endothelial expression of E-selectin and increased capillary density, as measured by CD31 positivity, in ischemic muscle treated with E-sel/AAV2/2. The primary mechanism by which we hypothesize E-selectin overexpression promotes angiogenesis is through recruitment of EPCs and perhaps other tissue repair cells to areas of ischemia and wound healing. In humans, EPCs were first characterized as CD34^+^/KDR^+^ cells but are now known to comprise a diverse population of cells expressing a variety of cell surface markers as they differentiate into mature endothelial cells (32–34). Homing of EPCs to areas of wound healing is mediated by SDF-1α-induced E-selectin/E-selectin ligand interactions on activated endothelium and circulating EPCs, respectively (15, 16). By increasing levels of vascular E-selectin in the ischemic tissue microenvironment, E-sel/AAV2/2 may help to potentiate and prolong the innate response to ischemia, thereby increasing post-natal vasculogenesis with subsequent benefit on skeletal muscle vascularity. Consistently, treatment with E-sel/AAV2/2 was associated with histological preservation of myofiber size and integrity which translated to improved exercise capacity on treadmill exhaustion testing.

While our findings demonstrated significantly enhanced EPC recruitment with E-sel/AAV2/2, there may be other mechanisms mediating the pro-angiogenic effects of E-selectin. Beyond homing of EPCs to areas of ischemia, our PCR array data show that local E-selectin overexpression induces a robust angiogenic response with upregulation of several growth factors, cytokines, cell adhesion molecules, and transcription factors. Of these, leptin (*Lep*) was the most significantly upregulated gene in ischemic muscle treated with E-sel/AAV2/2. Leptin is an adipokine that acts systemically as a satiety signal but has also been implicated in wound healing (35) and post-natal angiogenesis (36). Under physiological conditions, adipocytes in perivascular adipose tissue help to regulate vascular tone by releasing vasoactive molecules, including leptin, and these vasodilatory mechanisms may be impaired due to leptin-resistance associated with obesity (37). Additionally, perivascular cells expressing platelet-derived growth factor receptor α (PDGFRα) and PDGFRβ within skeletal muscle are leptin-producing and have been shown to induce production of VEGF-A by skeletal muscle cells (36). Other notable factors upregulated by E-sel/AAV2/2 included thymidine phosphorylase (platelet-derived endothelial cell growth factor, PD-ECGF) and placental growth factor (PGF) which have been shown to improve perfusion and exercise tolerance in rabbit hindlimb ischemia models (38, 39), as well as monocyte chemoattractant protein 1 (MCP-1) and integrin β3, which are critical for monocyte recruitment and arteriogenesis (40) and endothelial cell adhesion, migration, and endothelial tube formation, respectively (41). Future studies, including validation of angiogenic factor protein levels, are needed to better elucidate the mechanisms underlying therapeutic angiogenesis induced by E-sel/AAV2/2 gene therapy. Moreover, the beneficial effects observed on skeletal muscle histology and function warrant more detailed investigations into the effects of E-selectin on myocyte regeneration and mitochondrial metabolism.

Our preliminary safety data did not demonstrate a difference between mice treated with E-sel/AAV2/2, LacZ/AAV2/2 control vector, or PBS vehicle with regards to hematologic, hepatic, or renal function panels. However, D-dimer levels were elevated on POD 7 in E-selectin/AAV2/2-treated, as well as LacZ/AAV2/2 and PBS controls. These results likely indicate that neither E-selectin nor AAV2/2 vector alone was responsible for this effect. As we did not observe any evidence of microvascular thrombosis on histological examination, the supranormal D-dimer levels likely reflect recent surgical intervention. Of note, coagulation of the femoral vein in conjunction with the femoral artery was performed due to ease of the operation and may be clinically relevant as chronic venous insufficiency is a common comorbidity in patients with PAD (42). Regarding inflammation, PCR array demonstrated that E-sel/AAV2/2 modulated the inflammatory gene profile of ischemic limb tissue with an overall dampening or “cooling” effect on heatmap analysis. Although Mac-2^+^ macrophage infiltration was similar between E-sel/AAV2/2-treated and control tissue, the observed alteration in inflammatory cytokine and receptor gene expression may reflect improvement in the degree of ischemia in the tissue microenvironment, as well as direct downstream effects of E-selectin signaling and indirect paracrine actions of recruited cells. To account for the lag time between injection and transgene expression, our protocol called for administration of gene therapy at 4 and 2 days preoperatively, as well as on day of surgery. It should be noted that even with 3 separate administrations of gene therapy, these all occurred within a 4-day interval. In this short period, the immune system likely recognizes a single antigenic stimulus and does not have sufficient time to mount an antibody response. To this end, we found minimal recruitment of CD3^+^ T cells in either E-sel/AAV2/2-treated or LacZ/AAV2/2-treated ischemic muscle, indicating that neither E-selectin nor AAV2/2 vector induces a significant viral lymphocytic response, which is consistent with the well-documented low immunogenicity of AAV vectors (17).

The primary limitation of this study is the acuity of arterial disruption and subsequent ischemic injury. In our model, gangrene is induced in FVB mice by femoral artery and vein coagulation with additional administration of a nitric oxide synthase inhibitor to inhibit the endogenous vasodilatory response and increase tissue oxidative stress. Peak severity of tissue loss generally appears within 7–14 days with greatest therapeutic effect observed between 14 and 21 days. To develop a more chronic model of limb ischemia, other groups have used ameroid constrictors to gradually occlude the femoral artery (43, 44). With this technique, ischemia is less severe and associated with reduced inflammation and muscle necrosis compared to acute femoral artery ligation. However, the timing of ischemia onset in this model can be variable. Peak ischemia severity is reached within 14 days but may occur as soon as 3 days postoperatively with necrotic changes observed in BALB/c mice within 5 days (44). Perhaps more reflective of subacute ischemia, adapting this approach to FVB mice, which have intermediate sensitivity to ischemia, could prolong the duration of ischemia compared to C57BL/6 mice while improving the therapeutic window relative to BALB/c mice.

Importantly, our model does not account for comorbid smoking, hypertension, hyperlipidemia, or diabetes, which are common cardiovascular risk factors that also contribute to limb outcomes in PAD/CLTI. We also could not ascertain an independent effect of exercise therapy which is an essential component of management for PAD. In clinical practice, we envision using scoring systems such as the Wound, Ischemia, and foot Infection (WIfI) score (45), which incorporates tissue loss severity, hemodynamic parameters, and presence of infection, along with individual patient-specific factors such as disease etiology (i.e., diabetes, Buerger's disease) and vascular anatomy on angiography, to identify patients at risk of amputation who have exhausted traditional revascularization options and may benefit from angiogenic gene therapy.

While risk factor modification and revascularization remain central to treatment of PAD and CLTI, there is an unmet need for angiogenic therapies in patients with limited surgical revascularization options. Herein, we demonstrate the potential of E-sel/AAV2/2 gene therapy for reducing severity of tissue loss and improving perfusion and function of ischemic limbs. This preclinical study paves the way for studies in larger animal models and clinical trials in patients with inoperable CLTI.

## Data Availability Statement

The datasets presented in this study can be found in online repositories. The names of the repository/repositories and accession number(s) can be found below: https://www.ncbi.nlm.nih.gov/geo/, GSE201501.

## Ethics Statement

The animal study was reviewed and approved by the University of Miami Institutional Animal Care and Use Committee under protocol 19-163.

## Author Contributions

AR: study design, investigation, data analysis, and manuscript writing. YO: investigation, data analysis, and critical review. YL, CH, NL, and HS: investigation and critical review. RV-P: conceptualization and methodology. Z-JL and OV: conceptualization, methodology, manuscript editing, supervision, critical review, and funding acquisition. All authors contributed to the manuscript and approved the submitted version.

## Funding

This work was supported by grants from the National Institutes of Health [R01DK071084, R01GM081570, and VITA (NHLBl-CSB-HV-2017-01-JS)].

## Conflict of Interest

Z-JL and OV along with the University of Miami hold intellectual property of the AAV engineered E-selectin vector and have been licensed to Ambulero, Inc. The remaining authors declare that the research was conducted in the absence of any commercial or financial relationships that could be construed as a potential conflict of interest.

## Publisher's Note

All claims expressed in this article are solely those of the authors and do not necessarily represent those of their affiliated organizations, or those of the publisher, the editors and the reviewers. Any product that may be evaluated in this article, or claim that may be made by its manufacturer, is not guaranteed or endorsed by the publisher.
