# Supplementary figures and images for: E-Selectin/AAV2/2 Gene Therapy Alters Angiogenesis and Inflammatory Gene Profiles in Mouse Gangrene Model
Source: Front Cardiovasc Med. 2022 Jun 16;9:929466. doi: 10.3389/fcvm.2022.929466 (PMC9243393; doi:10.3389/fcvm.2022.929466)

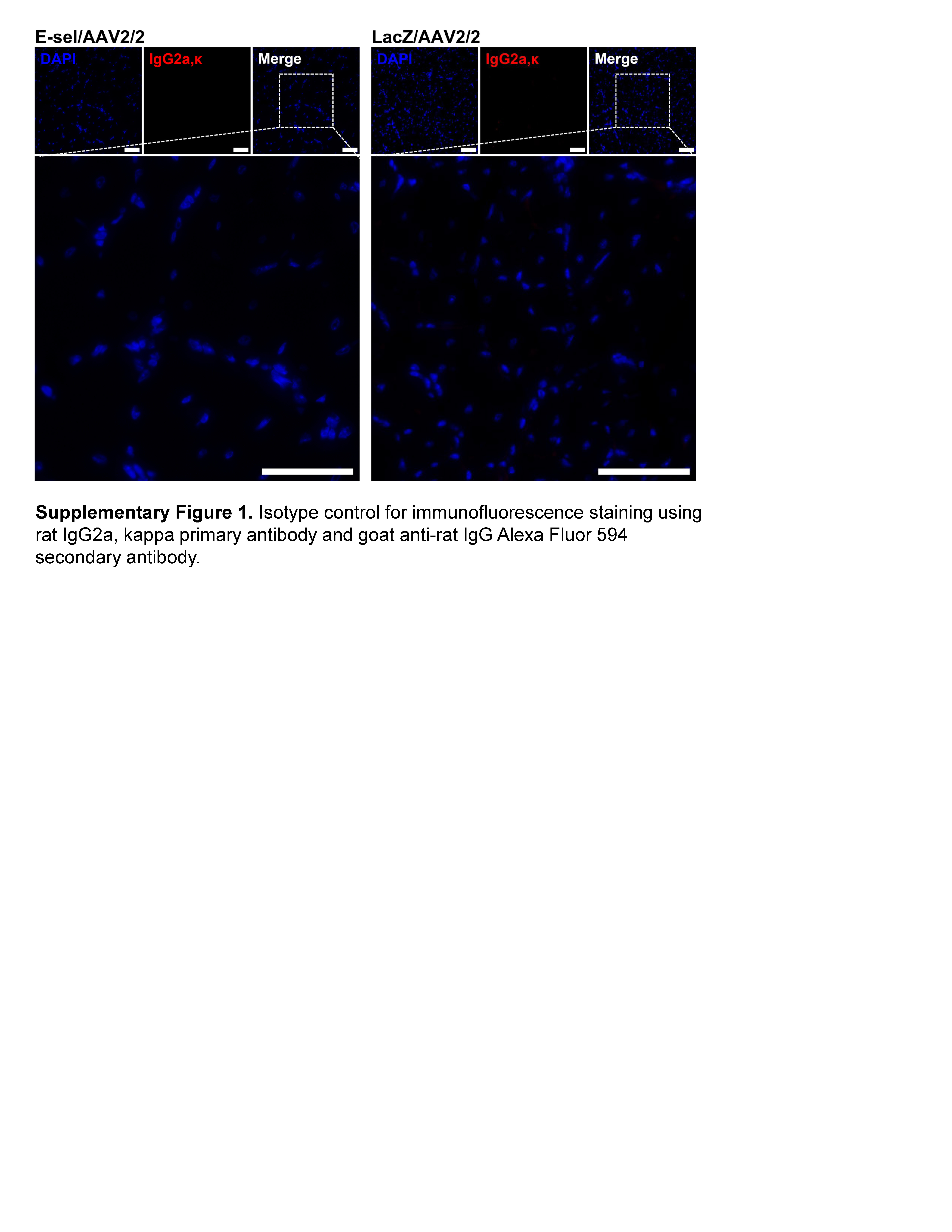

Supplement: Supplementary file 1 [file Image_1.jpg]

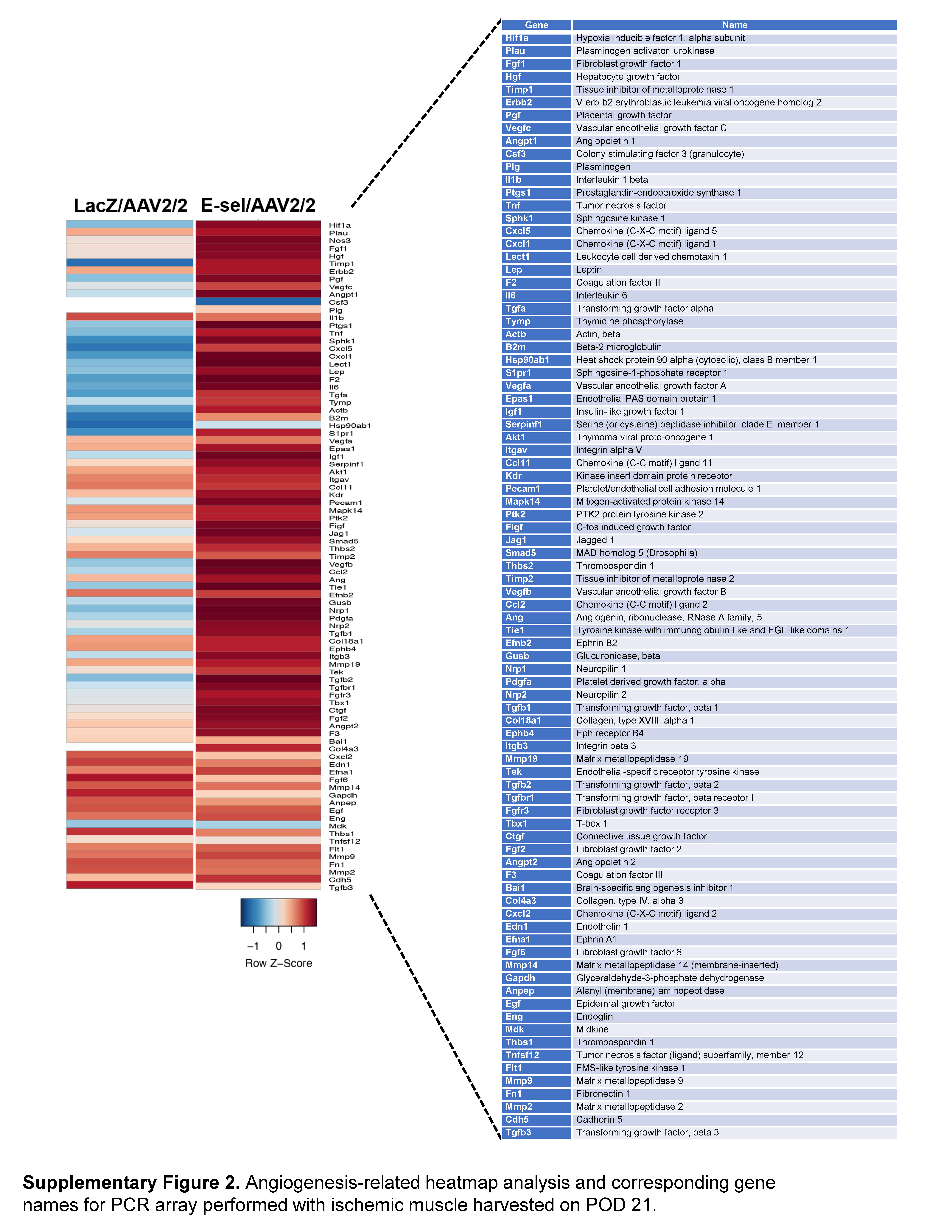

Supplement: Supplementary file 2 [file Image_2.jpg]

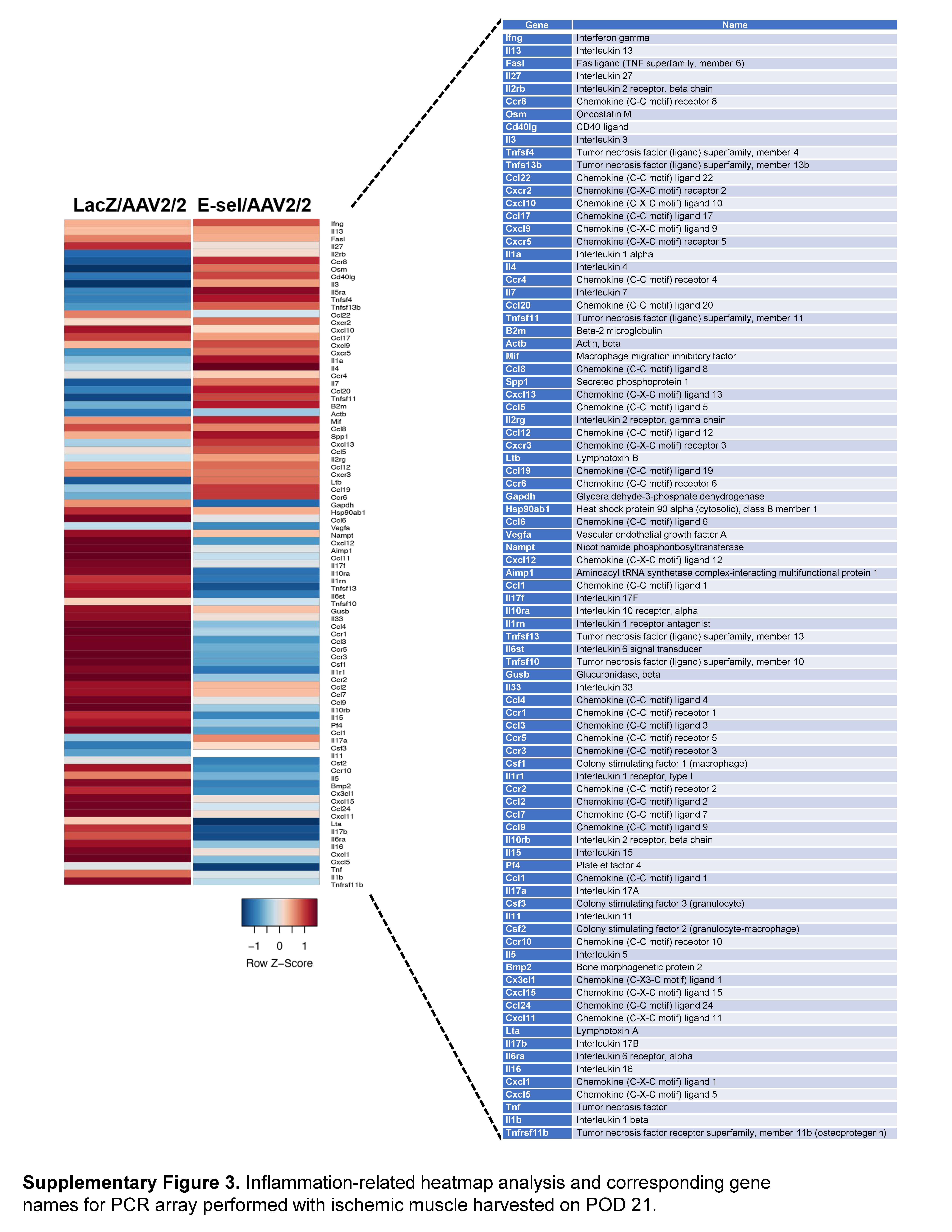

Supplement: Supplementary file 3 [file Image_3.jpg]
